# Virtual Reality as a Portable Alternative to Chromotherapy Rooms for Stress Relief: A Preliminary Study

**DOI:** 10.3390/s20216211

**Published:** 2020-10-30

**Authors:** Miguel A. Vaquero-Blasco, Eduardo Perez-Valero, Miguel Angel Lopez-Gordo, Christian Morillas

**Affiliations:** 1 Department of Signal Theory, Telematics and Communications, University of Granada, Calle Periodista Daniel Saucedo Aranda, s/n, 18014 Granada, Spain; miguelvaquero@ugr.es; 2 Research Centre for Information and Communications Technologies (CITIC), University of Granada, Calle Periodista Rafael Gómez Montero, 2, 18014 Granada, Spain; edu@ugr.es (E.P.-V.); cmg@ugr.es (C.M.); 3 Department of Computer Architecture and Technology, University of Granada, Calle Periodista Daniel Saucedo Aranda, s/n, 18014 Granada, Spain; 4 Nicolo Association, Churriana de la Vega, 18194 Granada, Spain

**Keywords:** virtual reality, EEG, emotions, stress, chromotherapy

## Abstract

Chromotherapy rooms are comfortable spaces, used in places like special needs schools, where stimuli are carefully selected to cope with stress. However, these rooms are expensive and require a space that cannot be reutilized. In this article, we propose the use of virtual reality (VR) as an inexpensive and portable alternative to chromotherapy rooms for stress relief. We recreated a chromotherapy room stress relief program using a commercial head mounted display (HD). We assessed the stress level of two groups (test and control) through an EEG biomarker, the relative gamma, while they experienced a relaxation session. First, participants were stressed using the Montreal imaging stress task (MIST). Then, for relaxing, the control group utilized a chromotherapy room while the test group used virtual reality. We performed a hypothesis test to compare the self- perceived stress level at different stages of the experiment and it yielded no significant differences in reducing stress for both groups, during relaxing (*p*-value: 0.8379, α = 0.05) or any other block. Furthermore, according to participant surveys, the use of virtual reality was deemed immersive, comfortable and pleasant (3.9 out of 5). Our preliminary results validate our approach as an inexpensive and portable alternative to chromotherapy rooms for stress relief.

## 1. Introduction

The possibility of creating virtual scenarios with fully immersive sensation and high realism is becoming more affordable and easier to develop due to the advances in virtual reality (VR) systems. However, traditional solutions are still widely used. For instance, chromotherapy is a type of light therapy that uses wavelengths in the visible region to treat disorders such as post-traumatic stress disorder (PTSD), panic, phobias [1,2] and is also used for stress relief [3]. This kind of therapy is applied in special needs education centers by means of chromotherapy rooms (comfortable spaces specially designed to facilitate relaxation by a combination of color, ambient lights and music) that are used as timeout rooms for children with behavioral issues. Nevertheless, the use of these rooms presents some downsides, such as the costly investment to accommodate a space for chromotherapy sessions, and the adaptation and maintenance of the room and its appliance (lights, sounds, insulation, etc.). The initial investment, according to commercial websites, is more than 3000 euros [4]. Moreover, the space required for the room cannot be used in any other context and its use for educational purposes may imply the isolation of the children that take part in these therapies. 

To overcome these drawbacks, in this paper we propose a VR application that can be executed in any commercial head mounted display (HMD). According to literature, virtual reality has been successfully applied in many fields, including phobia [5,6] and other disorders [7,8], rehabilitation [9,10,11], and areas such as learning [12,13,14], industry [15,16] and marketing [17], among others [18,19,20]. In most of the cited studies, the use of VR showed significant improvements, compared to traditional approaches. Additionally, VR is referred as “… an effective and equal medium …” when it does not constitute a better solution, as in [6]. Other studies have shown VR can help dealing with stress [21,22,23]. In addition, it is worth mentioning that a commercial VR device like the Oculus Quest HMD, that was used in this study, has a purchase price of 449 euros [24]. This is seven times less than the minimum necessary investment in a chromotherapy room. Furthermore, VR could also allow the implementation of chromotherapies with several participants at the same time, since only a headset per participant is required. These facts evidence the feasibility of using VR to create a relaxation environment. 

In order to carry out a stress assessment, several physiological markers have been validated in literature, namely electroencephalography (EEG) [25,26,27,28], electrocardiography (ECG) [29,30,31,32,33] or galvanic skin response (GSR) [33,34,35,36]. Among those biomarkers, EEG offers a similar stress detection performance compared to other established biomarkers, but a higher time resolution [37]. To evaluate our proposal and to compare it with the traditional alternative (chromotherapy rooms), we extracted the relative gamma from the EEG activity, as there are several studies that showed a relationship between the stress level and this biomarker [3,37,38]. The relative gamma is obtained using other EEG biomarkers (see the Methodology Section), also related to stress. It is noteworthy that under the same conditions, the relationship between the stress level and the relative gamma can present a direct or inverse proportion, depending on the participants [37,39,40]. Despite the reporting of this effect by several studies, the reason behind it is has yet to be clarified, though, it must be considered during processing.

The aim of this work is to propose a VR-based alternative to chromotherapy rooms, and to assess its effectiveness using EEG stress biomarkers. To fulfil a valid proposal, we followed a robust methodology where two groups of participants were conducted through a relaxation session. The first group experienced this session through our VR solution, whereas the second group utilized the classic alternative, a chromotherapy room. To the best of our knowledge, this is the first study that reproduced chromotherapy sessions via virtual reality, therefore, a direct comparison of the results of this research could not be carried out, as equivalent studies were not found in the literature. Nonetheless, in the Discussion Section, the main outcomes of this study are contrasted with papers that followed a similar methodology.

In conclusion, this study proposes an inexpensive and portable solution to incorporate chromotherapy sessions in any educative center, or even in new contexts, such as offices, hotels, hospitals or at home, that could make stress relief therapies more accessible. 

## 2. Methodology

### 2.1. Participants

Twenty healthy subjects voluntarily participated in the study (mean age, 24.20 ± 4.03 years). Only individuals without health issues and without mental disorders were considered for this experiment. Participants were recruited the week before the beginning of the study. They were asked to sign an informed consent form and not to take any relaxant nor stimulant the day before the test. Each participant underwent a single session that lasted approximately an hour. The complete data capture was accomplished in six days.

### 2.2. Experimental Process

**Figure 1:**
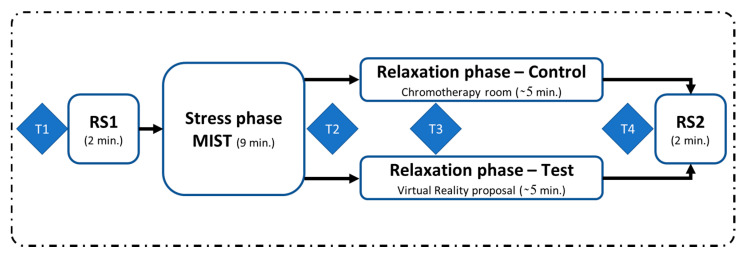
Time flow of the experimental process. RS1 and RS2 correspond to the resting state periods, where participants had to rest with their eyes closed. First, the participants completed RS1. Then, they went through the Montreal imaging stress test (MIST) test. The MIST phase was common for both groups and it consisted of a test with the aim of stressing the subjects. The next phase corresponded to relaxation, where participants were split into two groups. The control group was conducted to relaxation using a chromotherapy room, while the test group used our virtual reality proposal. Finally, a second resting state period, RS2, was completed. During the experiment, the participants filled several surveys (T1–T4) to report their perceived stress level. The experiment lasted approximately 18 min.

Before the beginning of the experiment, participants were equipped with a brain-computer interface (BCI). BCIs are typically closed-loop systems intended for the on-line analysis of the electrophysiological activity of the brain. In some cases, they can be used without feedback (i.e., open-loop) such us in the case of off-line detection of attention [41,42] or consciousness in non-communicative patients [43]. In these cases, they are reduced to the acquisition of brain activity for the posterior classification of cognitive states or previously-established codes. In this study we use the open-loop modality for the analysis of stress. The participants were randomly assigned to either the test group (TG: subjects S02, S04, S06, S09, S11, S13, S15, S17, S18, S20) or the control group (CG: subjects S01, S03, S05, S07, S08, S10, S12, S14, S16, S19). Once the participants were briefed on the different phases of the study and the tasks they had to complete, the experiment began. The whole process is illustrated in Figure 1.

First, the participants completed a resting state phase, where they were asked to remain calm for two minutes, with their eyes closed. Then, they were stressed using the Montreal imaging stress task (MIST), a test specifically designed to induce psychosocial stress [44]. Multiple studies in literature have endorsed the effectiveness of this test to elicit stress [45,46,47].

In next stage, participants from both groups completed a relaxation session. For the control group, the relaxation session was carried out in a chromotherapy room. These participants were sitting in a pouf inside a chromotherapy room and they were instructed not to move, in order to minimize the number of EEG artifacts. They were also asked not to close their eyes. Participants in the test group made use of our VR proposal during the relaxation session via an Oculus Quest HMD. They were seated in the same pouf used by the control group, but in an adjoining room. They were equipped with the HMD, which was previously configured. All the necessary conditions of the chromotherapy room were recreated in the virtual environment, which ensured that the outside environment could not interfere with the relaxing phase. These conditions included light and sound. The room temperature was also the same as in the chromotherapy room. In both scenarios, a loop of three specific colors (blue, magenta and green) was configured as ambient light. This procedure was suggested by an expert therapist, based on its empirical success in the special needs school where the study was conducted. The experiment ended with another two-minute eyes-closed resting state period. For both the control and test groups, the participants remained alone in their respective rooms during the relaxation phase and the resting state periods, and they were monitored from the outside.

During the experiment, the participants were asked to fill four surveys: one before RS1 (T1), another after the MIST (T2), one more 90 s after the beginning of the relaxation session (T3) and the last one at the end of the relaxation phase (T4), just before RS2. These surveys were used to gather the participants’ self-perceptions of stress during the study. Additionally, their EEGs were recorded, to obtain biomarkers to measure the participants’ stress levels. A separate survey was completed by the test group to evaluate their experience with our VR solution, in terms of comfort, immersion and general experience, on a scale from 1 to 5. 

### 2.3. Experimental Setup

The chromotherapy room used in this study was a 6 m^2^ chamber with white walls, floor and ceiling and a configurable ambient light used for relaxation sessions. It also had a beach poster on one of the walls and an IP video camera for security purposes. The room is used in the San Rafael special needs school (Granada, Spain) as a resting room for children with behavioral disorders. 

**Figure 2:**
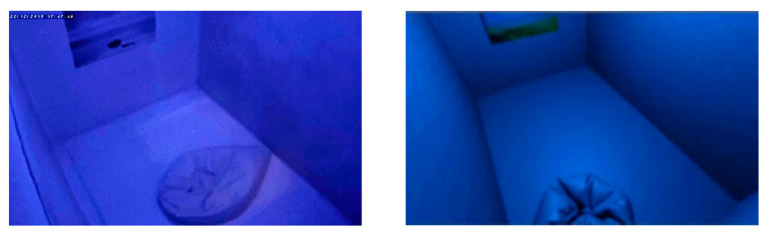
(**left**) The real chromotherapy room used with the control group and (**right**) our virtual room, simulating the same conditions.

The virtual chromotherapy room was developed using 2019 Unity software to recreate the features of the real chromotherapy room. To do so, the most similar textures were used on the walls and the floor, and the light was adjusted and positioned at the same angle, setting equal conditions of lighting and space compared to the reference room. These conditions included the ambient light colors and the transition time between the different colors during the ambient light loop (blue, magenta and green). Furthermore, the music track played during the virtual reality session was the same as the one used in the chromotherapy room sessions. The in-game view was also placed in the same position where the control group completed the session. To emulate the room comfort with high fidelity, the temperature was controlled and the same pouf was used. The real chromotherapy room and the one simulated using Unity are displayed in Figure 2.

Regarding the EEG setup, eight electrodes were placed at Fp1, Fp2, F7, F8, Fz, Cz, O1 and O2 positions of the International 10–20 system. These positions were selected according to a previous successful study on stress [37]. The electrodes were grounded and referenced to the left ear lobe. The EEG signals were acquired using the RABio w8 device (University of Granada, Spain) [38,48].

With respect to the MIST, it was implemented using a graphical interface developed in MATLAB R2016a (MathWorks, Natick, NA, USA). To complete the MIST, the participants were comfortably seated in a chair and they were asked to move only one hand to complete the task via the computer’s touchscreen. The MIST test had a duration of 9 min, including a 3-min training period. During training, the participants completed a series of arithmetical operations (additions, subtractions, multiplications and divisions) without time limit. Over the test period, the participants had to complete the same kind of calculations but each of them had an imposed time limit. This time limit was displayed to the participants as a progress bar on top of the screen. After each calculation, a second progress bar briefed the participant with his/her success rate during the task. Additionally, one of the technicians in charge of the experiment entered the room on three occasions during the test period in order to verbally put pressure on the participant. For a complete description of the MIST protocol, refer to [44].

The surveys used to gather the participants’ self-perceived stress levels were an adaptation of the perceived stress scale (PSS), where the time needed to complete the questions was minimized. In these surveys, participants answered with a number, according to the level of stress they felt, where 1 was the minimum and 5 was the maximum (“Is your current stress level higher or lower than the last time we asked? What is that level from 1 to 5?”).

### 2.4. Signal Processing

First, we concatenated the EEG recordings from the different blocks of the experiment, including only the central minute of the two resting state periods. Next, we applied a second order zero-phase shift bandpass Butterworth filter, from 1 to 50 Hz, to the EEG signals, and we also applied a notch filter at 50 Hz to remove electric coupling [3,37]. 

Subsequently, we performed a spectral analysis. The EEG signals were divided into two-second epochs without overlapping, and epoch channels above 100 µV were zeroed. Each epoch was then detrended and z-scored. The power spectral density (PSD) was calculated in different frequency bands (see Table 1) and then averaged across EEG channels in order to be jointly analyzed.

Next, we obtained the relative gamma (RG), a biomarker linked to stress, as the power ratio between gamma and slow rhythms (alpha and theta), as indicated in Equation (1). We extracted only this biomarker due to its proven feasibility in stress detection [37,38,40].

(1) $$\mathrm{RG}=\frac{{\mathrm{Power}}_{\mathrm{Gamma}}}{{\mathrm{Power}}_{\mathrm{Alpha}-\mathrm{Theta}}}$$

The RG was then smoothed using a moving average filter of 20 samples, and resampled to obtain arrays of the same length (480 samples) for all subjects. This array length corresponds to the 16-min duration of the experiment (the duration of the experiment was about 18 min but only the central minute of the resting state periods was considered for processing), divided in two-second epochs, as presented by Equation (2).

(2) $$480_{\mathrm{epochs}}=\frac{16_{\min}\cdot \;60_{s/\min}}{2_{s/\mathrm{epoch}}}$$

Finally, through visual inspection, subjects whose relative gamma was inversely proportional to the stress level felt were detected, in order to be processed separately. The RG of those participants was inverted to be averaged with the data from subjects whose relative gamma was directly proportional to their stress level.

### 2.5. Statistical Analysis

The grand-average across all participants and the standard error of the mean (SEM) were estimated for the relative gamma. A 6th degree polynomial was fitted to the mean RG for both the control and test group to easily compare the stress response of both groups. We then calculated the Pearson correlation coefficient (PCC) between the fitted curves to assess their similarity.

We also computed the mean and standard deviation of the self-perceived stress level (SPSL) of the participants in the CG and TG, using the answers to the surveys T1–T4. The non-parametric Wilcoxon signed-rank test was applied to compare the stress perceived by participants in both groups. This test was employed because the data did not pass the normality Lilliefors test (*p*-value > 0.05). The Wilcoxon test is used to assess whether the population mean ranks differ in two datasets. The test was also applied to check if there were significant differences in the stress perceived by participants during the different phases of the experiment. For all statistical tests, the level of significance, alpha, was set to 0.05 (*α* = 0.05).

Finally, we obtained the mean and standard deviation for the VR user experience survey answers. These answers were used to evaluate the level of immersion, comfort and general experience perceived by the participants in the test group.

## 3. Results

Due to issues during signal acquisition, data from three participants (S03, S05 and S14, all belonging to the control group) were discarded. Regarding artifact removal, a maximum of 9 percent of the epochs were rejected for a participant.

**Figure 3:**
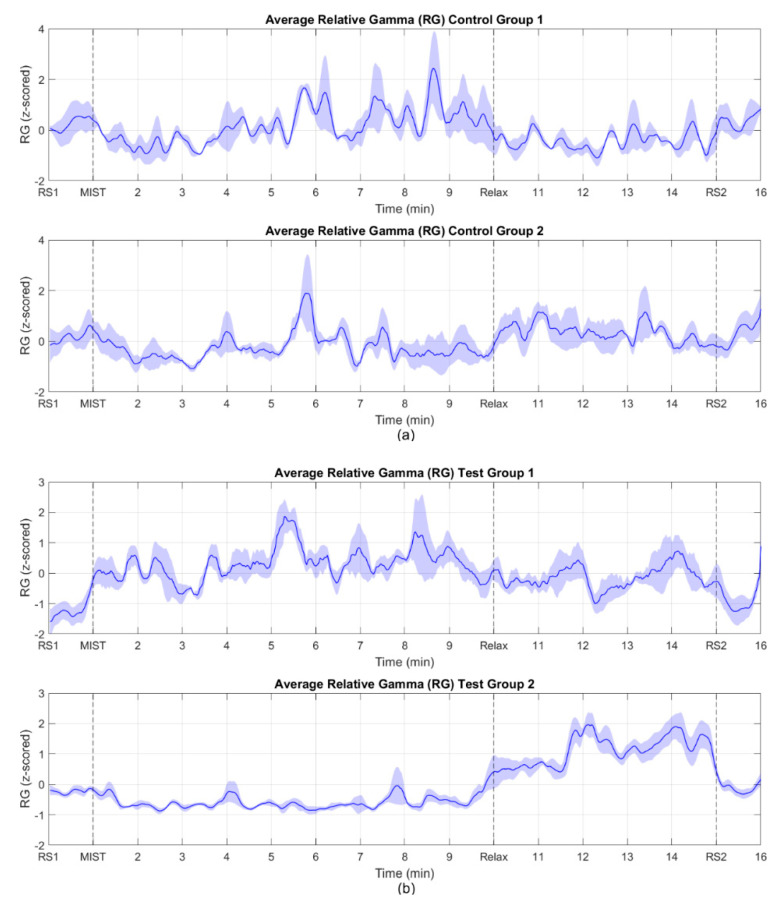
Time evolution of the average relative gamma in the regions of interest. Group 1 (upper) corresponds to the subjects whose response was directly proportional to the stress level experienced. In Group 2 (bottom), the biomarker response was inversely proportional to their stress level. RS1 and RS2 match the central minute of the resting state blocks. MIST indicates the beginning of the stress session (3 min of training and 6 min of task). Relax indicates the beginning of the relaxing session (5 min). The shaded areas represent the standard error of the mean (SEM). (**a**) control group. (**b**) test group.

As mentioned in the previous section, there are studies that found the relative gamma is inversely proportional to the stress level, while in other studies, this proportion was identified as direct. In our experiment, 7 out of 17 of the participants’ relative gamma values were directly proportional to the stress level felt, whereas the rest of the participants (10 out of 17) showed an inversely proportional relationship. Considering this, we classified them as “Group 1” (subjects S02, S07, S08, S11, S16, S17 and S20), and “Group 2” (subjects S01, S04, S06, S09, S10, S12, S13, S15, S18 and S19) depending on whether they exhibited a directly or inversely proportional response, respectively. Figure 3a,b shows the average across subjects of the time evolution of the processed stress biomarker for the control and the test groups.

**Figure 4:**
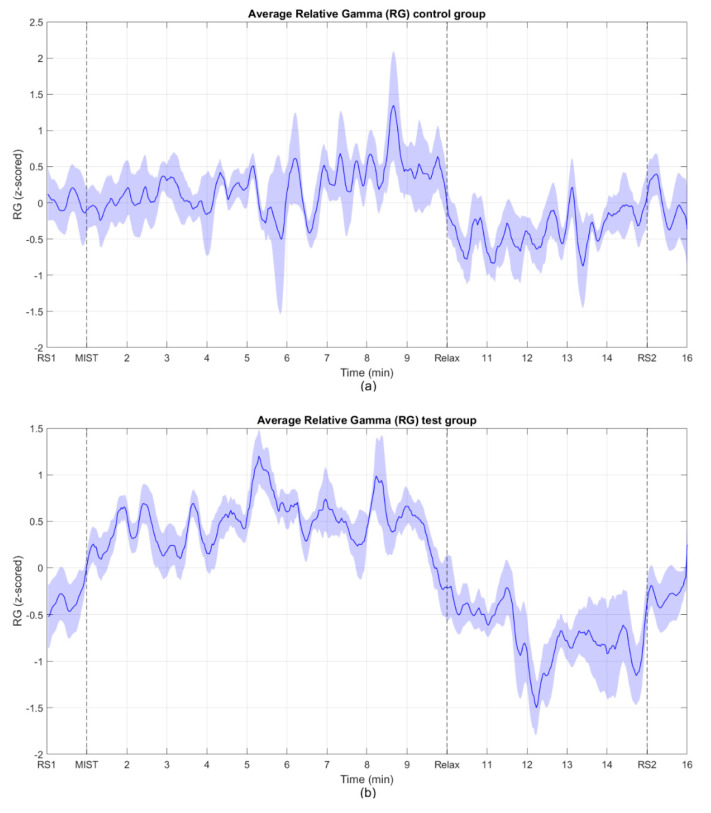
Time evolution of the average relative gamma in the regions of interest. Both Group 1 and 2 are considered in the average. The response of the subjects in Group 2 was inverted before computing the average. RS1 and RS2 correspond to the central minute of the resting state blocks. MIST indicates the beginning of the stress session (3 min of training and 6 min of task). Relax indicates the beginning of the relaxing session (5 min). The shaded areas represent the standard error of the mean (SEM). (**a**) control group. (**b**) test group.

Figure 4 shows the mean across all subjects of the relative gamma for the control group and the test group. To combine the results from Group 1 and Group 2, we first inverted the relative gamma of the participants that belonged to Group 2 and then we calculated the average.

**Figure 5:**
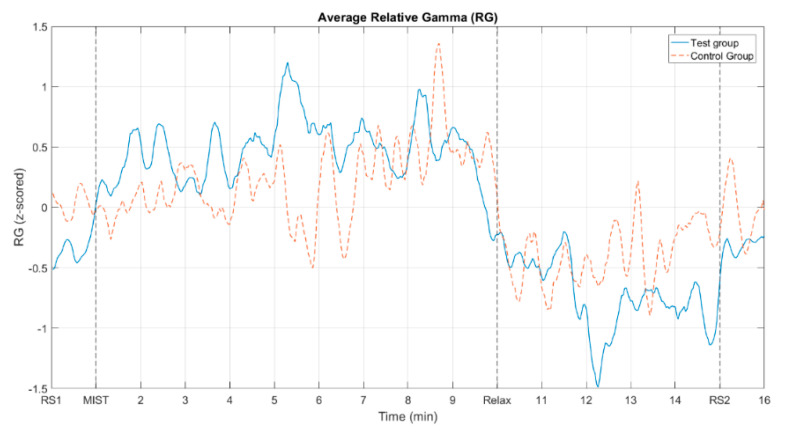
Average relative gamma for both the control group and the test group. The solid line corresponds to the test group. The dashed line corresponds to the control group.

Figure 5 displays the mean across all subjects of the relative gamma for both the control and the test group. This figure represents jointly the graphs from Figure 4 after removing the shaded areas for a clearer visualization.

**Figure 6:**
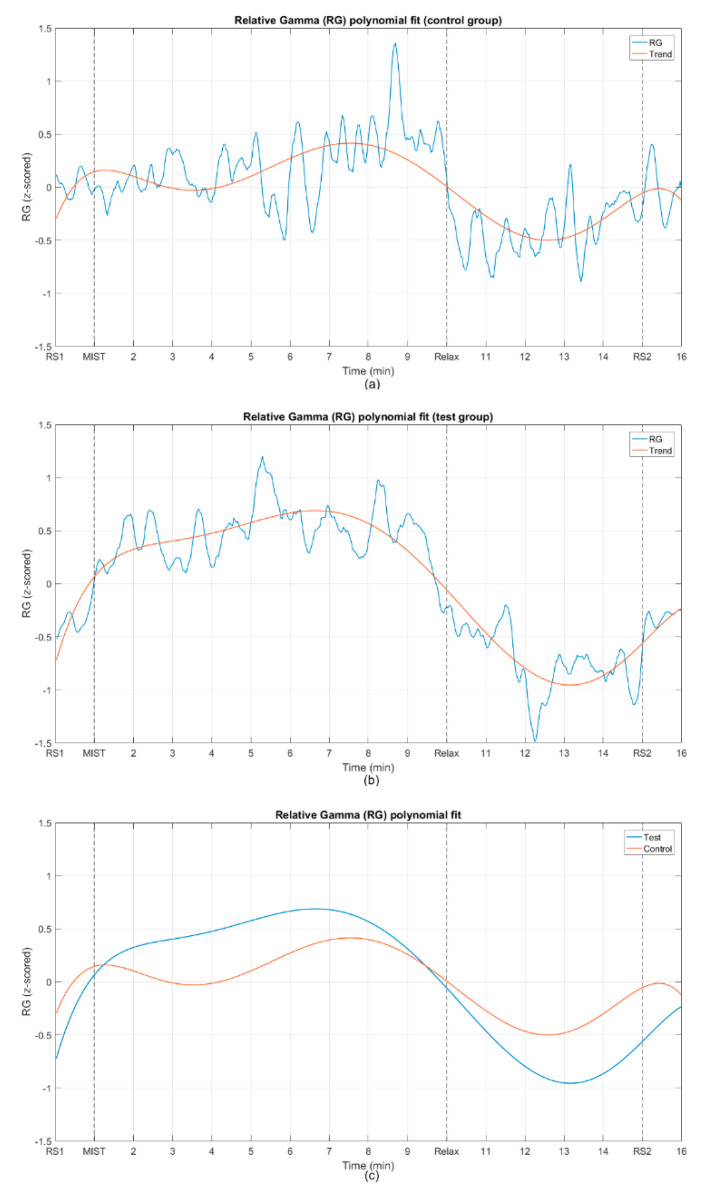
Fits of the average Relative Gamma with a 6th degree polynomial for (**a**) the control group and (**b**) the test group. (**c**) Comparison of the polynomial fits of the relative gamma for both groups.

Figure 6 illustrates the fitting of the relative gamma averaged across participants with a 6th degree polynomial, for both the control group and the test group.

Table 2 presents Pearson’s correlation coefficient (PCC) calculated between the average relative gamma across participants for the control and test groups, and between the polynomial fits, including the lower and upper bounds, for a 95% confidence interval for each coefficient. 

**Figure 7:**
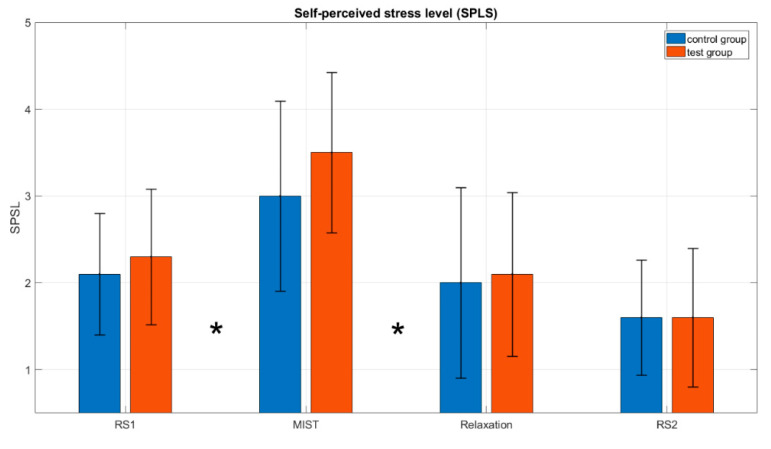
Average SPSL across participants obtained from surveys at the four moments of interest T1: before Resting State 1 (RS1); T2: just after the MIST; T3: 90 s after the beginning of the relaxation phase and T4: before Resting State 2 (RS2). Black lines represent the standard deviation of the mean. Asterisks (*) indicate, for both groups, a statistically significant difference between the SPSL at RS1 compared to the MIST, and the SPSL at the MIST in comparison to the relaxation phase (Wilcoxon signed rank test, *p*-value < 0.05). When comparing the SPSL at the same phase for the two groups, no significant differences were found for any of the phases (α = 0.05).

Figure 7 shows the average across participants of the SPSL at four instants of interest (T1, T2, T3 and T4, see Figure 1), for the control group and the test group.

The results of the different Wilcoxon signed rank tests that were performed are shown in Table 3 and Table 4. Table 3 displays the *p*-values of the tests, comparing the SPSL responses during the different phases of the experiment, for both the control and test groups. Table 4 shows the *p*-values of the tests carried out to compare the SPSL responses between the two groups of subjects.

**Figure 8:**
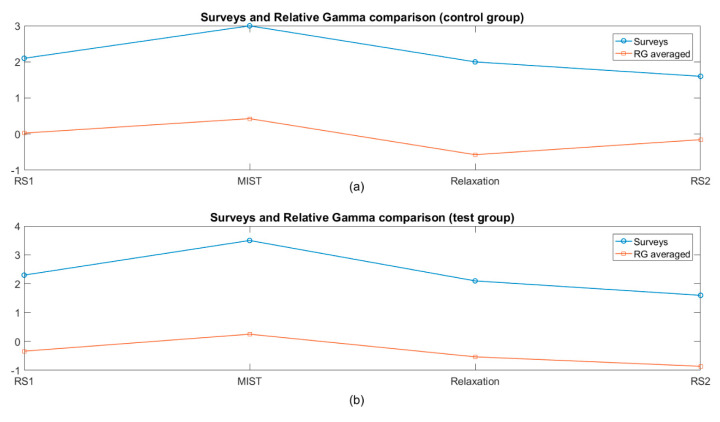
Average SPSL and relative gamma across participants at four moments of interest (T1, T2, T3, and T4). Relative gamma at the four instants was obtained as the average of the RG values of a minute centered in each moment. The labels RS1, MIST, Relaxation and RS2 correspond to the phases of the experiment where the surveys T1 to T4 were conducted. Units were not included in the vertical axis due to the different magnitudes of the curves. (**a**) the control group and (**b**) the test group.

Figure 8 shows the average across participants of the SPSL and the relative gamma at four instants of interest (T1, T2, T3 and T4), for the control group and the test group. Pearson’s correlation coefficient (PCC) was calculated between the average SPSL and relative gamma, across participants at the four instants of interest (T1–T4), for both control and test groups, resulting in values of 0.73 and 0.99, respectively.

**Figure 9:**
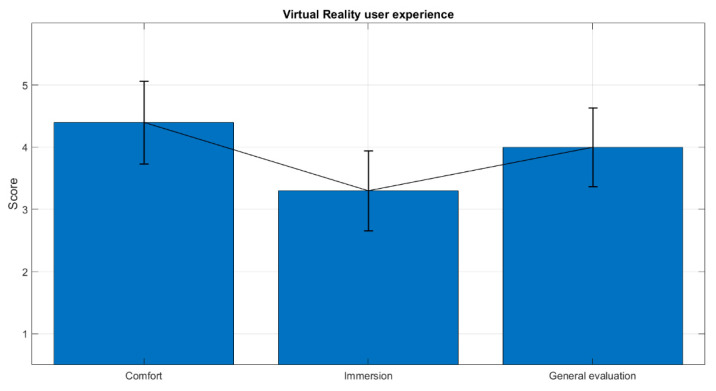
Average VR user experience across participants. Black lines indicate the standard deviation of the mean. The X axis represents the three aspects evaluated by the participants during the VR user experience survey, namely, levels of comfort and immersion and general evaluation of the VR system. The Y axis indicates the scores participants gave to the aspects aforementioned that ranged from 1 (minimum) to 5 (maximum).

Finally, Figure 9 depicts the average VR user experience across participants of the test group.

## 4. Discussion

The aim of this work was to assess the feasibility of VR as an alternative to chromotherapy rooms for stress relief. After conducting the same relaxing program in both the chromotherapy room and using our VR solution, the preliminary results support that our approach offers a similar performance compared to the room, and thus represents a handy alternative. Despite the availability of other stress biomarkers (e.g., cortisol or heart rate), previous works have shown evidence of the suitability of the RG as a reliable stress biomarker and they have highlighted its temporal resolution [3,37,38]. As explained previously, the magnitude of the RG can be directly or inversely proportional to the stress level experienced. Despite some works obtaining a direct response [3], others found an inverse behavior [39,40], or even both trends in the same study [37]. This is the reason why participants were split into Group 1 and Group 2 (in Group 1, RG increased during MIST and decreased during Relax and vice-versa in Group 2) as shown in Figure 3. Although this behavior has been considered during signal processing, the rationale behind it is out of the scope of this work. The dataset contained the data from 20 participants (480 samples, 16 min × 30 samples per min) and just one independent variable (RG). Although the results are enough to justify our findings, a higher number of participants would likely have yielded a better correlation coefficient and smaller confidence intervals.

Figure 4 shows the average RG across participants for the test and control groups, where the responses of the subjects from Group 2 were inverted. These curves show evidence of a positive trend of the relative gamma during the MIST phase and a negative trend during the relaxation phase. As expected, the RG levels of the two resting state blocks at the beginning and the end of the experiment were similar. It is worth mentioning that in both the control and the test groups the RG dropped to its minimum after approximately two minutes from the beginning of the relax block (circa minute 11 and 12 for control and test group, respectively). As mentioned in the Introduction Section, as far as we know, there are no other studies that implemented virtual reality-based chromotherapy sessions. The most similar papers in the literature studied the stress relief effect of actual chromotherapy sessions [3,37]. In these studies, the reported evolution of the RG is similar to the one we obtained here, strengthening the methodology followed in our approach. Once the minimum stress level was reached, the RG slightly increased during the rest of the relaxation phase, Once the minimum stress level was reached, the RG slightly increased during the rest of the relaxation phase, (possibly due to boredom or some unintentional cognitive activity) until it reached a level at the final resting state that approximately matched that of the first resting state period. These findings suggest that a maximum of approximately two minutes is necessary in both the VR application and the chromotherapy room to reach the minimum level of stress. Since a typical relaxation program, such as the one used in this study, lasts for 5 min or more (see Figure 1), our results suggest that this duration could be largely reduced. Special needs schools could benefit from this finding, since children who had to leave the classroom for a chromotherapy session could be back sooner. Finally, it should be taken into account that due to the use of a moving average filter to smooth the signals, the start of the drop and the rise of the RG do not exactly match the start of their respective phases, what was expected according to the works previously referenced. Considering that the filter span used was 19 points, including the center sample (the 9 previous and 9 subsequent points), and each sample represents a two-second epoch, the obtained values, including the start of the drop or the maximum value during the MIST phase, are shifted by around 20 sec with respect to the theoretical values.

When we compare the average relative gamma of the control group and the test group, they present similar time–course dynamics (see Figure 5). The behavior of this biomarker for the two groups is analogous to the one reported by previous studies on stress assessment using relative gamma [3,38]. This is more evident in Figure 6c, where the 6th degree polynomials fitted to the relative gamma curves are compared. We utilized curve fitting to reduce the statistical fluctuations and inherent variability of the EEG, and then estimate the correlation between the relative gamma of both groups. The value of Pearson’s correlation coefficient that we obtained provides evidence of the similarity of the stress curves of both the control and test group (see Table 2).

In relation to the analysis of the SPSL (see Figure 7), we found significant differences between T1 and T2, and between T2 and T3, for both the control and the test group, as indicated by the asterisks in the figure. This proves that all participants increased their stress levels during the MIST and decreased them during the relaxation phase. This was expected and it provides evidence that the experiment was properly designed and that it caused the required levels of stress and relaxation.

A visual inspection of Figure 7 shows that the levels of stress indicated by the RG bars are approximately the same for both the test and control groups. Furthermore, since the chromotherapy room is a traditional method used for stress relief, we enquired ourselves how good this approach was compared to our VR solution. To assess this issue, we conducted non-parametric tests to compare the SPSL in the control and the test groups at each of the four instants of interest (T1, T2, T3 and T4). We did not find significant differences for any of the four instants (see Table 2). In summary, the results presented in Figure 7 and Table 2 suggest that the level of stress achieved in a chromotherapy room are similar to those obtained in the VR environment.

Finally, the great similarity between SPSL and the relative gamma curves (see Figure 8), as well as the high correlation values (namely 0.73 and 0.99, for the control and test groups respectively) confirm the appropriateness of the RG as a reliable biomarker for the stress assessment analysis performed in this study.

## 5. Conclusions

In this work, we propose the use of VR as an alternative to chromotherapy rooms for stress relief. For this purpose, twenty participants were evenly stressed using a well-established methodology (the MIST test) and then conducted through the same relaxation program, half by means of VR and the other half by means of a chromotherapy room. The stress levels were assessed through a self-perceived stress level survey at four key moments in the experiment. Additionally, the EEG activity was recorded and the relative gamma was computed to extract a reliable stress biomarker. If we compare both approaches, our VR solution presents many advantages over chromotherapy rooms. First, the costs associated with VR are much lower than those associated with investments and maintenance of standard chromotherapy rooms. Also, our proposal could be implemented on any commercial off-the-shelf device, thus it could increase the availability and ubiquity of chromotherapies. This makes our solution suitable for new environments which could benefit from this kind of therapy, such as offices, hotels, sport centers, etc., and bring their stress reduction support level closer to schools that cannot afford a chromotherapy room. Even regular people could access these relaxing programs by running an application on a head mounted display at home. Finally, as shown in Figure 9, the use of our VR proposal was deemed pleasant, comfortable and immersive by the participants. In summary the obtained results validate our VR approach as an alternative to chromotherapy rooms for stress relief. For future studies, we propose the use of other more immersive and customizable variants of VR such as interactive VR 360 videos, as they could potentially improve the performance of standard VR and chromotherapy rooms. In view of the accurate matching of the relative gamma curves with the SPSL scores (in terms of visual inspection of Figure 8 and correlation of 0.77 and 0.99 for the control and the test group), we suggest to increase the number of times that participants answer the SPSL survey. This would enable the regression of the stress level on the relative gamma, thus gaining new insights from the time–course dynamics of stress with higher temporal resolution. Finally, future studies should consider the combination of EEG with other biomarkers, such as GSR or heart rate (HR) to examine if they provide a better understanding of the stress relief properties of chromotherapy sessions.

## Figures and Tables

**Table 1:** Frequencies of the bands where the PSD was estimated.

Frequencies Band	Range (Hz)
Delta	1–4
Theta	4–8
Alpha	8–13
Betha	13–25
Gamma	25–45

**Table 2:** Pearson’s correlation coefficient (PCC) and lower and upper bounds for the 95% confidence interval, between the RG for the control and test groups, and between the polynomial fits.

Pair	CI Low	PCC	CI Up
RG control RG test	0.54	0.60	0.65
RG control fit RG test fit	0.87	0.89	0.91

**Table 3:** *P*-values obtained with the Wilcoxon signed rank tests to compare the SPSL responses of the subjects during the experiment. RS1 and RS2 correspond to responses given in the first and second resting state periods, respectively. The (*) indicates significant differences between the two datasets. For all tests, the significance level was 0.05 (α = 0.05).

Group	RS1-MIST	MIST-Relax	Relax-RS2
Test group	0.0020 *	0.0117 *	0.1250
Control group	0.0313 *	0.0156 *	0.2500

**Table 4:** *P*-values obtained with the Wilcoxon signed rank test to compare the SPSL responses given by the test and the control groups. RS1 and RS2 correspond to responses given in the first and second resting state periods, respectively.

RS1	MIST	Relax	RS2
0.7500	0.1875	0.8379	0.8125
